# Descriptive Analysis of Anatomical Location and Metabolic and Microbiological Factors in Diabetic Foot (DF) Treated at a DF Specialty Tertiary Care Hospital With a Multidisciplinary Approach

**DOI:** 10.7759/cureus.80690

**Published:** 2025-03-16

**Authors:** Amit R Burande, Meeta A Burande, Siddhi N Powar, Tanmay U Vora

**Affiliations:** 1 Anatomy, D. Y. Patil Medical College, Kolhapur, IND; 2 Orthopaedics and Diabetic Foot Surgery, Surya Hospital, Kolhapur, IND; 3 Pharmacology, D. Y. Patil Medical College, Kolhapur, IND; 4 Diabetology, Surya Hospital, Kolhapur, IND; 5 Clinical Research, Surya Hospital, Kolhapur, IND; 6 General Medicine, Surya Hospital, Kolhapur, IND

**Keywords:** amputation, diabetic foot, diabetic foot ulcers, surgery, treatment outcome

## Abstract

Background and aims

The outcome of a diabetic foot ulcer (DFU) may be affected by many factors, including the clinical anatomy of the ulcer, metabolic control, the presence of complications, and infections. This study evaluates the effect of metabolic factors, the clinical anatomy of the ulcer at presentation, and antibiotic appropriateness on treatment outcomes in terms of amputation in patients with diabetic foot (DF).

Materials and methods

This is a cross-sectional observational study involving data collected from patients with IPD diagnosed with type 2 diabetes mellitus and DFU at a tertiary care DF specialty hospital in Maharashtra, India.

Results

Data from a total of 106 patients were included for analysis. Fifty-two patients healed without amputation while 54 underwent either minor or major amputation. Patients who did not require amputation had a statistically significant higher incidence of cellulitis, ulcers at the heel and lateral malleolus, and ulcers classified as Wagner grade 1 or 2. In contrast, factors significantly associated with amputation included lower weight and BMI, multiple ulcers at presentation, ulcers involving the second, third, fourth, or fifth toe, midfoot, or medial malleolus, ulcers graded 3, 4, or 5, and additional surgery performed during the same hospital admission. The most commonly collected specimen was pus, followed by tissue and bone. The most frequently isolated microorganisms were *Escherichia (E.) coli* and *Klebsiella*. All microorganisms were sensitive to Piperacillin-Tazobactam and matched the initially prescribed antibiotic, which was chosen as per the hospital antibiogram. Angiotensin-converting enzyme inhibitors (ACE-I)/angiotensin receptor blockers (ARBs) were prescribed more frequently in the non-amputation group while insulin use was higher in the amputation group, although there was no significant difference in the use of metabolic drugs between the two groups.

Conclusion

The anatomical location of the wound, advanced grade, number of ulcers, additional procedures, nutritional status, and the presence of Coagulase-negative* Staphylococcus aureus* in tissue are significant predictors of amputation.

## Introduction

The rise of diabetes in India is alarming, increasing from 7.7% in 2016, as per the Indian Council of Medical Research (ICMR)-India Diabetes (INDIAB) [1], to 9.3% in 2018, as per NCD NNMS [2]. Diabetic foot ulcer (DFU) is a complication of diabetes, with a global prevalence of 6.3%, ranging from 1.5% to 16.6% depending on the region and country. The lifetime risk of developing DFU in a diabetic patient is 25% [3]. It is a leading cause of disability and a significant healthcare burden [4,5].

The management and treatment outcomes of DFUs may be influenced by several factors, including the clinical anatomy of the ulcer, metabolic control, the presence of complications, and infections [2].

Locally, factors such as the clinical anatomy of the ulcer, duration and number of ulcers, location, grading, severity, presence of vascular insufficiency and infection, foot deformity, and the need for offloading and surgical procedures may all play a role in determining treatment outcomes [6-10].

Among metabolic predictors, advanced age, male gender, smoking [11], higher-than-normal BMI, duration of diabetes, poor metabolic control, and the presence of neuropathy are associated with poor outcomes [8]. Additionally, appropriate antibiotic selection is crucial for managing polymicrobial-resistant infections [12,13]. A comprehensive antibiogram, including data on bacteriology and antibiotic sensitivity, can facilitate the early selection of appropriate antibiotics in a healthcare setting [14].

Thus, metabolic control, along with additional risk factors, including complications, the clinical anatomy of the ulcer [6], and antibiotic resistance, may influence hospital length of stay and treatment outcomes in terms of mortality and wound healing, with or without amputation [10].

Currently, there is a scarcity of studies in the Indian population evaluating the effect of metabolic factors, clinical anatomy of ulcers, and antibiotic appropriateness on treatment outcomes in managing DFUs among patients diagnosed with type 2 diabetes mellitus (T2DM).

## Materials and methods

Research objects

This is a cross-sectional observational study involving inpatients diagnosed with T2DM and DFUs at a tertiary care DF specialty hospital in Maharashtra, India, between January 2023 and August 2024, following approval from the Institutional Ethics Committee. Patients were included in the study if they were admitted to the hospital and met the selection criteria. Informed consent was obtained from all patients before surgery.

Selection criteria

Patients with T2DM (as per the Research Society for the Study of Diabetes in India (RSSDI) 2024 criteria) with foot ulcers classified according to International Working Group on the Diabetic Foot (IWGDF) guidelines [15] were admitted to the hospital for wound management.

Exclusion criteria

Pregnant patients were excluded from the study.

Sample size

The sample size was calculated to be 106, based on an anticipated proportion of the population with DFUs at 16%, an absolute precision of 5%, a 95% confidence limit, and a margin of error of 7%.

Research content and data collection methods

Patients meeting the study criteria underwent data collection regarding their medical history, general characteristics, and clinical features.

General Subject Characteristics

General characteristics included age, height, weight, BMI, and gender (male/female). The duration since the detection of type 2 diabetes was categorized as <5 years, 5-10 years, and >10 years. Blood sugar control was assessed in known diabetic patients based on adherence or non-adherence to medication. Medical history covered smoking, alcohol and tobacco use, hypertension, amputation, and peripheral artery disease (PAD) [16]. Family history was recorded, including details of diabetes in the mother, father, brother, or sister.

Clinical Anatomy Characteristics

Clinical characteristics included the duration of foot ulcers, categorized as <2 weeks, 2-4 weeks, and >4 weeks. Ulcer location and the presence of multiple ulcers were recorded. Foot ulcers were graded based on the Wagner classification, which consists of five grades (grades 1 through 5) [17].

Tests Conducted to Classify T2DM With and Without Infected Foot Ulcers

Blood sugar levels at admission were recorded, along with routine investigations, including CBC (Hb, N, L, ESR), blood urea level (BUL), Sr. Creatinine, HbA1C, and GFR. Doppler ultrasonography of the lower extremity arteries was performed to assess atherosclerosis severity, categorized as mild stenosis (0%-49%), moderate stenosis (50%-75%), and severe stenosis (>75%) [18].

Hospitalization Parameters Collected Until Discharge

During hospitalization until discharge, data collected included the initial antibiotic prescribed, the type of surgeries performed, the need for additional surgery, and the duration of hospital stay.

Bacteriological Characteristics of Diabetic Foot Infections

Bacteriological analysis of diabetic foot infections (DFIs) included identifying the type of isolated bacteria and performing culture sensitivity testing for antibiotic resistance. Initial antibiotic therapy was prescribed based on IWGDF guidelines. During surgery, samples were collected and sent to the lab for bacterial identification and antibiotic sensitivity testing. The appropriateness of the initial antibiotic was assessed using the obtained report and categorized as YES if the prescribed antibiotic was sensitive to the isolated bacteria or NO if it was resistant.

Surgical Characteristics

Based on the outcome at the time of discharge, patients were divided into two groups: “WITHOUT AMPUTATION,” consisting of those who did not require amputation, and “AMPUTATION,” which included patients who underwent amputation. The AMPUTATION group was further classified into Minor amputation (single or multiple toe amputations) and Major amputation (below-knee or above-knee amputations). The results were recorded in the master dataset and analyzed. Quantitative variables were analyzed using the chi-square test while qualitative variables were assessed using the t-test, with a significance level of P ≤ 0.05.

## Results

A total of 106 patients were included in this study between January 2023 and August 2024, with a mean age of 59.54 ± 11.68 years. Males (79; 74.5%) were predominant over females (27; 25.5%) (Table 1). All parameters were not significantly different between the two groups except for BMI, which was significantly higher in patients without amputation (23.45 ± 3.79) compared with those who required amputation (25.37 ± 4.11, P = 0.01).

**Table 1:** Demographic characteristics and medical history of patients in this study CHD: coronary heart disease

Criteria	Study Patients
Age	59.54 ± 11.68
Male	79 (74.5%)
Female	27 (25.5%)
Height (m)	1.64 ± 0.08
Weight (kg)	65.77 ± 13.42
BMI	24.39 ± 4.05
Duration of DM	11.91 ± 8.48
Family history of DM	35 (33.01%)
Hypertension	59 (55.66%)
Dyslipidemia	47 (44.33%)
CHD	17 (16.03%)
Osteomyelitis	19 (17.92%)
Heart failure	20 (18.86%)

**Figure 1:**
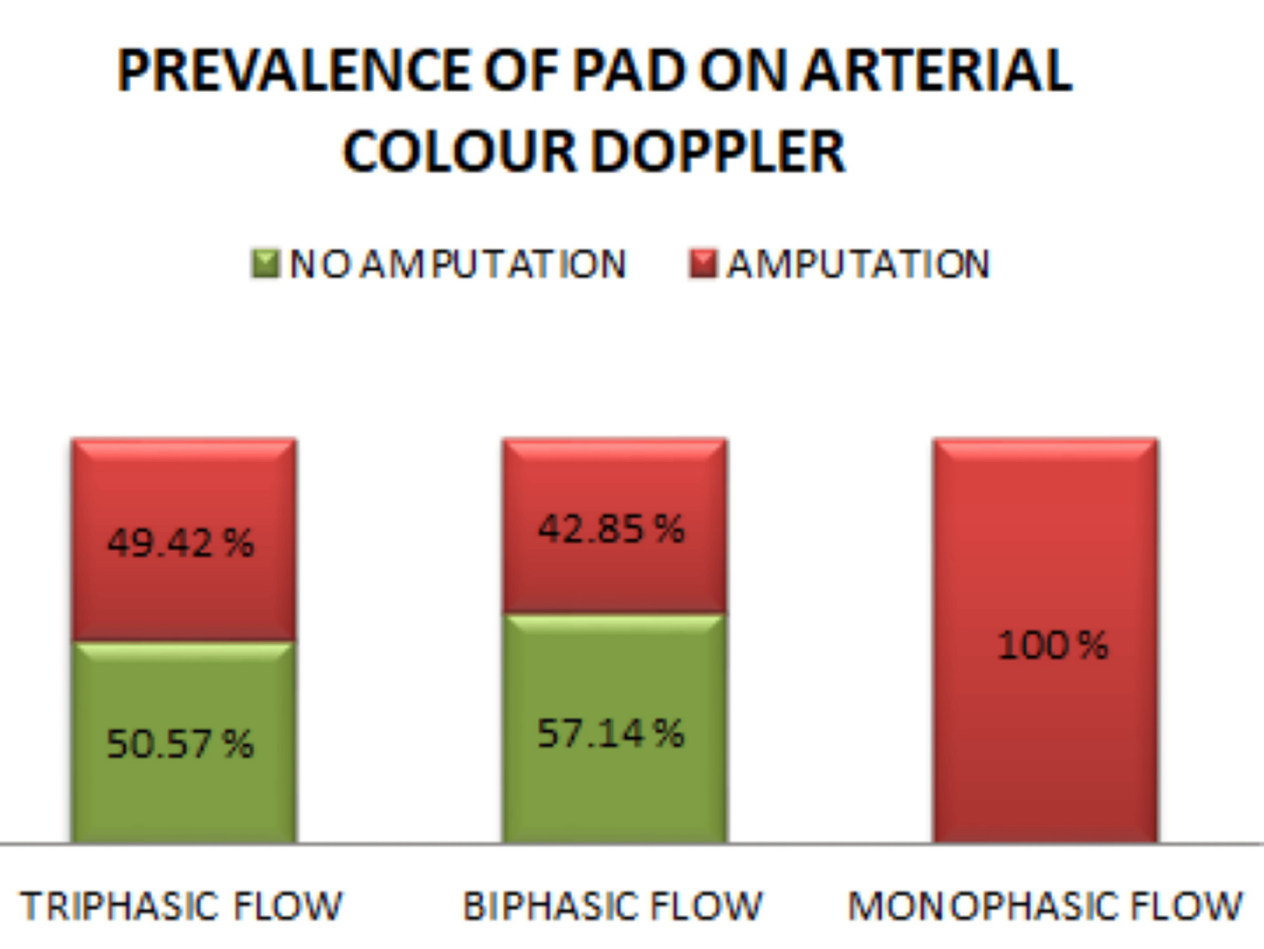
Prevalence of PAD on arterial color Doppler PAD: peripheral artery disease

Peripheral artery disease (PAD) was assessed using color Doppler imaging. Among the patients, 87 (82.07%) had triphasic flow; of these, 44 (50.57%) did not require amputation while 43 (49.42%) underwent either major or minor amputation. Fourteen patients (13.20%) had biphasic flow, with 8 (57.14%) not requiring amputation and 6 (42.85%) requiring amputation. Five (4.7%) patients had monophasic flow, and all of them underwent amputation (Figure 1). We prescribed Trental (pentoxifylline) 400 mg twice daily to all patients. Additionally, cilostazol was prescribed at 50 mg twice daily for patients with mild atherosclerotic changes and 100 mg twice daily for those with moderate or severe atherosclerotic changes. Patients were advised to undergo angiography or angioplasty as recommended by the vascular surgeon. However, among these patients, 10 underwent angiography, of whom 7 had mild atherosclerosis, 2 had moderate atherosclerosis, 1 had severe atherosclerosis, and none opted for angioplasty.

**Figure 2:**
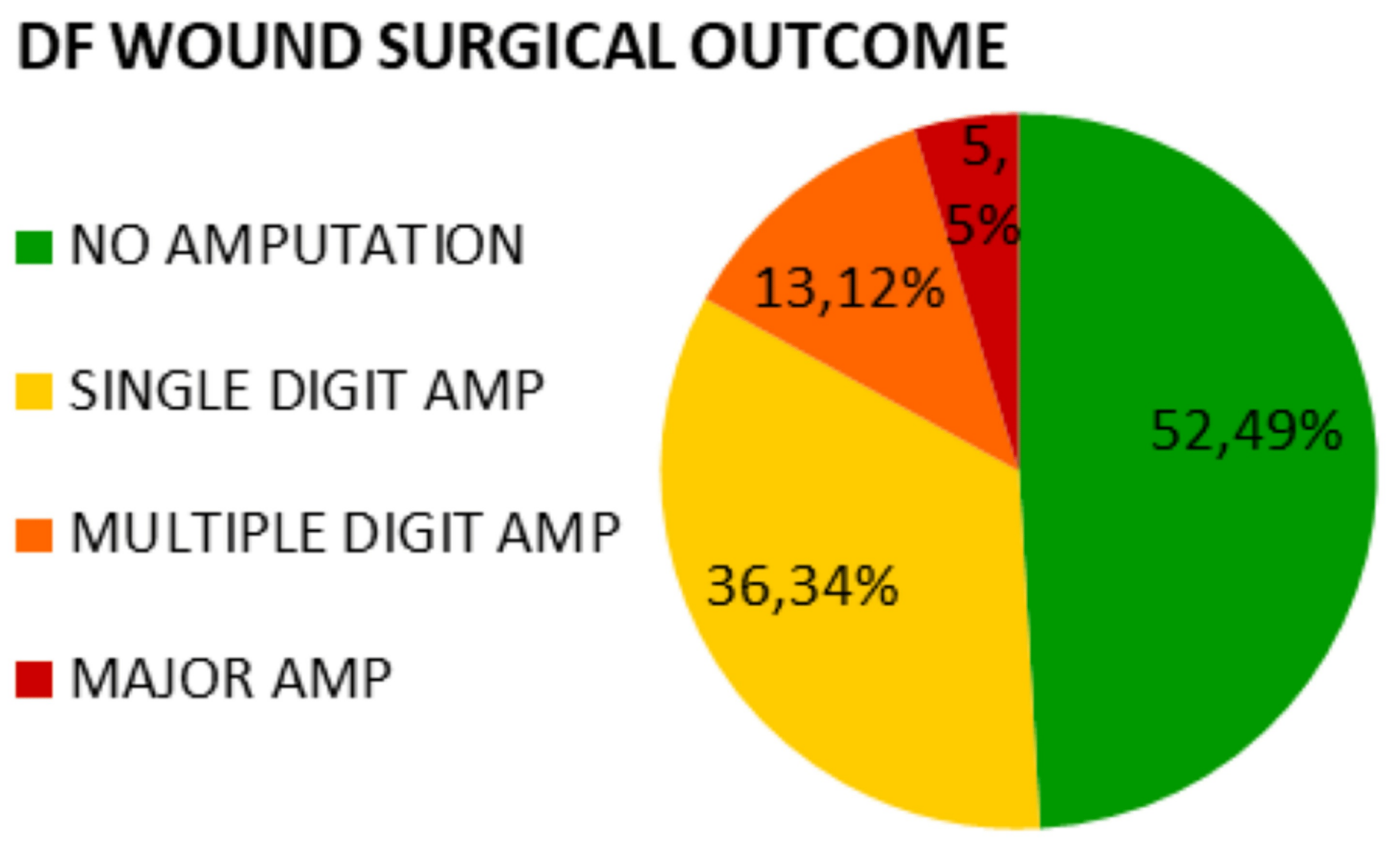
Surgical outcome of diabetic foot (DF) wounds

Patients underwent various procedures based on the clinical profile of their ulcers. Regarding the surgical outcome of DF wounds, 52 (49%) patients required only debridement and healed without amputation. Among the remaining patients, 36 (34%) underwent single-digit or ray amputation, 13 (12%) required multiple-digit amputation, and 5 (5%) required below-knee or above-knee amputation (Figure 2).

Anatomical location of the ulcers

The duration of ulcers was categorized into three groups: <2 weeks, 2-4 weeks, and >4 weeks, and there was no significant difference between the two patient groups in ulcer duration. Similarly, there was no significant difference in the history of previous amputation among different groups. The incidence of multiple ulcers was significantly higher in the amputation group as compared with patients who healed without amputation.

**Figure 3:**
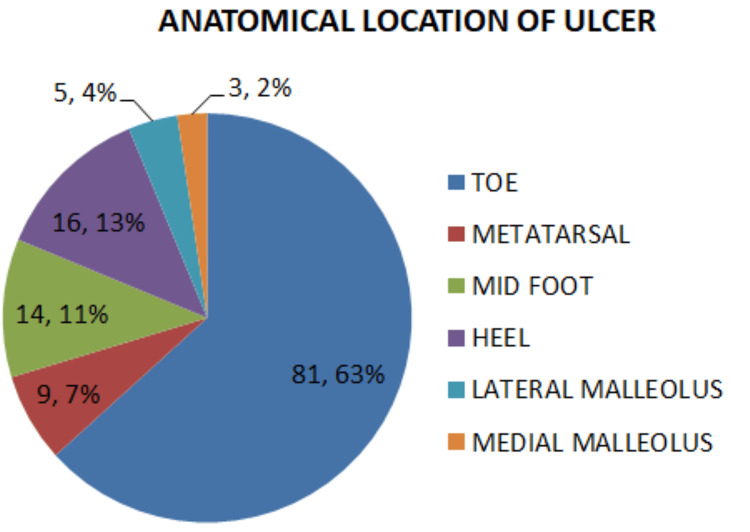
Anatomical location of ulcer

Out of 106 patients, left extremity involvement was more common than right extremity involvement, though there was no significant difference between the two groups. Ulcers located at the great toe, metatarsal head, and lateral malleolus did not show a significant difference in outcomes between the groups. Patients with cellulitis and ulcers at the heel usually healed without amputation and had significantly higher chances of foot salvage. In contrast, ulcers at the second, third, fourth, and fifth toes, medial malleolus, and midfoot had significantly higher chances of amputation and required significantly more additional surgeries during hospitalization (Table 2, Figure 3).

**Table 2:** Ulcer duration and anatomical location NAMP: No Amputation; AMP: Amputation

CRITERIA		NAMP	AMP	TOTAL	P-VALUE
Ulcer duration	<2	19	22	41	0.66
	2–4	19	13	32	0.16
	>4	14	19	33	0.36
Previous amputation		16	22	38	0.28
Multiple ulcers		9	22	31	0.008
Right foot		24	23	47	0.86
Left foot		32	33	65	0.99
1^ST^ toe		10	14	24	0.41
2^ND^ toe		4	13	17	0.02
3^RD^ toe		3	10	13	0.04
4^TH^ toe		2	11	13	0.01
5^TH^ toe		2	12	14	0.01
Metatarsal		5	4	9	0.69
Midfoot		3	11	14	0.03
Heel		12	4	16	0.02
Lateral malleolus		4	1	5	0.15
Medıal malleolus		0	3	3	Highly Significant
Cellulitis		22	7	29	0.001
Additional surgery		1	7	8	0.03

We categorized patients based on Wagner’s grading system, which distinguishes between acute infections and deep ulcers. The grading system is referenced in our study and is defined as follows: Grade 0 - Intact skin; Grade 1 - Superficial ulcer (affecting only the skin, no deeper tissue involvement, e.g., cellulitis); Grade 2 - Ulcer extending into deeper structures (tendons, ligaments, joint capsule, but no bone involvement); Grade 3 - Ulcer with deep infection (involvement of bone, abscess formation, or osteomyelitis); Grade 4 - Partial gangrene (localized to toes or forefoot); Grade 5 - Extensive gangrene (involving the entire foot, often requiring amputation).

Our findings indicate a significant difference between the groups. Patients whose limbs were salvaged without amputation were primarily in Grade 1 and Grade 2 (superficial ulcers) while deep ulcers were a significant risk factor for amputation (Tables 3, 4).

**Table 3:** Wagner’s grading

	Grade 0	Grade 1	Grade 2	Grade 3	Grade 4	Grade 5
Amputation	0	3	7	23	19	2
No Amputation	0	21	24	6	1	0
TOTAL	0	24	31	29	20	2
P-value	--	0.000018	0.000112	0.000509	0.000012	Highly Significant

**Table 4:** Significantly different predictors between treatment outcomes

ANATOMICAL FACTORS	NO AMPUTATION	MINOR / MAJOR AMPUTATION
Location of ulcer	Heel cellulitis	2^ND^, 3^RD^, 4^TH^, 5^TH^ toe, midfoot, medial malleolus, multiple ulcers, additional surgery
Grading of ulcer	G0, G1, G2	G3, G4, G5

Bacteriological characteristics of diabetic foot infections

**Figure 4:**
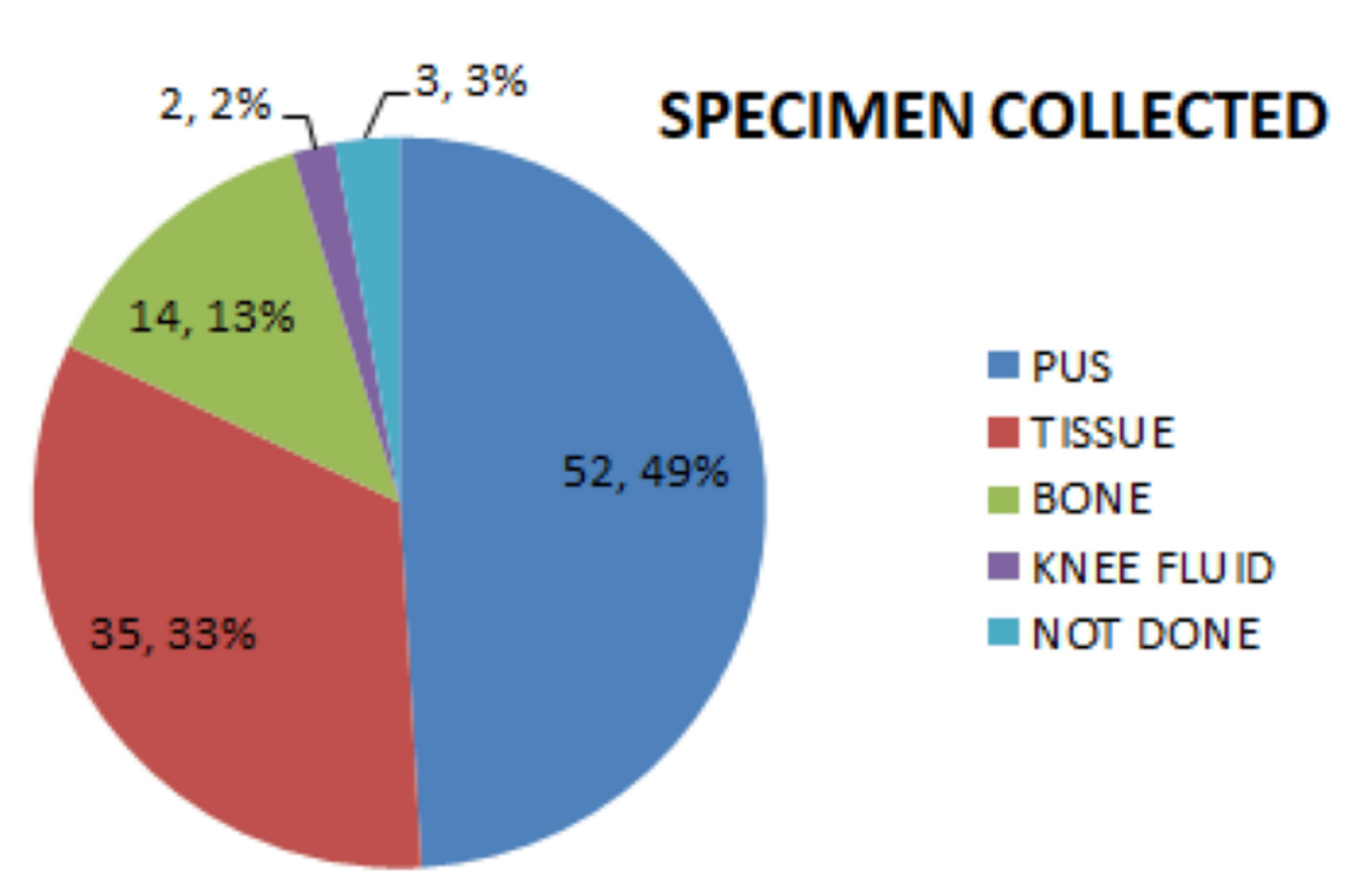
Specimen collected during surgery

Among all patients, pus was the most commonly collected specimen compared with other sample types (Figure 4).

In the non-amputation group, *E. coli *was the most commonly isolated microorganism, followed by *Klebsiella*. The most frequently collected specimen was pus, followed by tissue. *Citrobacter freundii* was found only in bone, while *Proteus vulgaris* was found only in tissue. *Citrobacter koserii* and *Enterobacter* were isolated only from pus. In the amputation group, pus remained the most commonly collected specimen, and *Klebsiella *was the most frequently isolated microorganism. Coagulase-positive *Staphylococcus aureus* was found only in tissue, while *Citrobacter koserii*, *Enterobacter*, and *Proteus vulgaris* were found only in pus (Table 5). Coagulase-negative *Staphylococcus aureus* was significantly more common in tissue samples from the amputation group (P = 0.02).

**Table 5:** Bacteriological characteristics of wounds NAMP: No Amputation; AMP: Amputation; Coag.: Coagulase; SA: Staphylococcus aureus; C.: Citrobacter; P.: Proteus

ISOLATED MICROORGANISM	PUS		TISSUE		BONE		KNEE FLUID		NOT DONE		TOTAL
	AMP	NAMP	AMP	NAMP	AMP	NAMP	AMP	NAMP	AMP	NAMP	
E. Coli	10	5	2	5	1	2	0	0	0	0	25
Klebsiella	6	4	5	5	4	1	0	0	0	0	25
Coag. −ve SA	3	4	6	1	0	0	0	0	0	0	14
Coag. +ve SA	0	1	1	2	0	0	0	0	0	0	04
Acinetobacter	0	0	0	1	0	1	0	0	0	0	02
Pseudomonas	3	2	0	0	0	0	0	0	0	0	05
C. Freundii	0	0	0	0	0	1	0	0	0	0	01
C. Koserii	1	1	0	0	0	0	0	0	0	0	02
Enterobacter	1	1	0	0	0	0	0	0	0	0	02
P. vulgaris	1	0	0	1	0	0	0	0	0	0	02
Not done	0	0	0	0	0	0	0	0	2	1	03
No growth	4	5	2	4	2	2	0	2	0	0	21
Total	29	23	16	19	07	07	00	02	02	01	106

Drugs

ACE-Is/ARBs were prescribed more frequently in patients who healed without amputation while insulin use was higher in the amputation group. However, there was no significant difference in the use of other metabolic drugs between the two groups (Table 6).

**Table 6:** Routine medicines ACE-I/ARB: Angiotensin-converting enzyme inhibitor/angiotensin receptor blocker; CCB: calcium channel blockers; DPP4: dipeptidyl peptidase; SGLT2: sodium-glucose cotransporter-2; GLP-1: glucagon-like peptide 1; ANA: antinuclear antibody

	TOTAL	NAMP	AMP	P-VALUE
Cardiovascular drugs				
ACE-I/ARB	40	24	16	0.08
CCB	27	14	13	0.736
Beta-blockers	10	6	4	0.466
Nitrates	3	1	2	0.58
Diuretics	31	18	13	0.232
Statins	47	26	21	0.249
Antiplat.	61	30	31	0.976
Anticoag.	2	1	1	0.978
Anti-diabetic drugs				
Vogli	14	6	8	0.62
Piogli	5	3	2	0.62
Met	54	25	29	0.56
SU	41	23	28	0.43
DPP4 Inhi	36	16	20	0.5
SGLT2	16	10	6	0.24
Insulin	62	26	36	0.08
GLP 1 ANA	1	1	0	NC

## Discussion

After analysis of the research outcome, we found that patients who required amputation had a significantly lower BMI compared with patients who did not require amputation. Similar results were mentioned in a meta-analysis carried out by Lin et al. in 2020, which found that patients with lower BMI are more likely to undergo DFU-related amputations [19]. In the present study, there was no significant difference in the duration of DM and the duration of ulcers between the two study groups.

Among the various comorbidities in study patients, hypertension was the most commonly observed. Similar results were found in studies conducted in Ethiopia [20], the Netherlands [9], and Greece [21]. Bone involvement due to osteomyelitis was present in 17.92% of study patients. A study conducted by Le et al. also reported approximately similar results (20.9%) [10] while a study by Hicks et al. found that osteomyelitis occurred in 71.7% of study patients [21].

In this study, PAD was assessed using color Doppler to evaluate blood flow in arteries. The results showed that the majority of patients had triphasic flow, indicating mild atherosclerosis, similar to findings in a study conducted in Vietnam [10]. Patients with abnormal Doppler ultrasound findings were managed based on the severity of atherosclerotic changes. Angiography and possible angioplasty were considered based on the vascular surgeon’s recommendation. 

In this study, patients underwent different surgical interventions depending on the severity of the ulcer. Surgical options included debridement and, in severe cases, minor or major amputations. Out of 106 patients, 49% required only debridement, 46% underwent minor amputation, while the need for major amputation was only 5%. In comparison, a study at All India Institute of Medical Sciences (AIIMS) Jodhpur showed that 19.6% of patients underwent debridement, 59% required minor amputation, and 22% needed major amputation [22]. Similarly, a study in Brazil reported a major amputation rate of 11.7% [23].

After an analysis of the anatomical location of ulcers, involvement of the left extremity was more as compared with the right in all patients, in contrast to a study conducted in Uganda in 2023, which showed that the right foot was affected more [24]. The majority of patients had ulcers at the toes, similar to studies in Vietnam by Le et al. and in Catalonia by Bundo et al. [10,25].

The severity of ulcers was assessed by Wagner’s grading, which shows that a higher grade of foot ulcer leads to slower healing or the need for major amputation. Similar results were seen in studies conducted in Ethiopia [20], Korea [26], and China [27]. Overall, the most commonly isolated bacteria were E. coli and Klebsiella. A study by Burande et al. in India showed that Klebsiella was the most commonly isolated bacteria among patients with DF [14], and in both studies, pus was the most commonly collected specimen. Meanwhile, a study in Vietnam showed that Staphylococcus was the most frequently isolated bacteria [10].

The present study shows 100% appropriateness of antibiotics, probably due to the predefined antibiogram of the hospital being followed [14], while a study by Bekele and Chelkeba [20] reported 49.35% appropriateness of initial antibiotics. This may highlight the need for a hospital antibiogram for initial antibiotic selection.

A study conducted by Lapray et al. found that patients exposed to ARBs were positively associated with wound healing [28]. In our study, ACE-I/ARB was prescribed more in patients who did not require amputation while insulin use was higher in the group of patients who required amputation. Meanwhile, a study conducted in New York mentioned that outcomes in high-risk critical limb ischemia may be improved by the use of ACE-I/ARB [29].

In the present study, 2 (1.8%) patients died during treatment at the hospital due to septicemic shock. The first patient had additional risk factors of hypertension (HTN) and chronic kidney disease (CKD), while the second patient had renovascular disease (RVD) and HTN. A study carried out at AIIMS Jodhpur reported a 7% mortality rate [22], while a study in the UK by Phyo et al. showed a 38% mortality rate [30].

This is a hospital-based study, which may introduce selection bias, as it primarily includes inpatients rather than a broader real-world population. Patient selection was influenced by local policies, ulcer severity, and the need for inpatient care, which may limit the generalizability of our findings to outpatient or community-based settings.

Additionally, the study has a lower statistical power due to the pre-determined sample size, and there is an inherent margin of error in the findings. Some patients may not have undergone all recommended investigations due to various constraints.

While we aimed to provide a comprehensive analysis, factors such as variability in ulcer characteristics, patient adherence to treatment, and resource availability may have influenced the outcomes.

Despite these limitations, our study offers valuable real-world insights into the treatment and management of diabetic foot patients in the Indian context. The findings contribute to existing knowledge and may serve as a foundation for future larger-scale studies to further explore treatment outcomes in a more diverse patient population.

## Conclusions

Overall, the amputation group had a significantly lower BMI, which may highlight the importance of nutrition and better diabetes control. Multiple ulcers were more frequent in the amputation group. Among all patients, left extremity involvement was more common as compared to the right. Ulcers located on the heel or those associated with cellulitis were less likely to require amputation while ulcers on the toes, midfoot, and medial malleolus were more often linked to amputation. Multiple ulcers at presentation, the need for additional surgery during hospitalization, and severe ulcer grading were identified as risk factors for amputation. *Klebsiella* and *E. coli *were the most commonly isolated bacteria, and pus was the most frequently collected specimen. The high appropriateness of antibiotic use may be attributed to adherence to a predefined antibiogram.

In summary, wound location, ulcer severity, number of ulcers, need for additional procedures, nutritional status, and the presence of bacterial infections in tissue samples appear to be significant predictors of amputation.

## References

[1] India State-Level Disease Burden Initiative Diabetes Collaborators (2018). The increasing burden of diabetes and variations among the states of India: the Global Burden of Disease Study 1990-2016. Lancet Glob Health.

[2] Mathur P, Leburu S, Kulothungan V (2022). Prevalence, awareness, treatment and control of diabetes in India from the Countrywide National NCD Monitoring Survey. Front Public Health.

[3] Piran N, Farhadian M, Soltanian AR, Borzouei S (2024). Diabetic foot ulcers risk prediction in patients with type 2 diabetes using classifier based on associations rule mining. Sci Rep.

[4] Zhang X, Li Q, Zhou X, Xu Y, Shu Z, Deng H (2024). Risk factors for amputation in diabetic foot ulcers: a retrospective analysis [Retracted]. Int Wound J.

[5] Pfannkuche A, Alhajjar A, Ming A (2020). Prevalence and risk factors of diabetic peripheral neuropathy in a diabetics cohort: register initiative “diabetes and nerves.”. Endocr Metab Sci.

[6] Raha A (2023). Personal hygiene and SGLT2i. Chronicle of Diabetes Research and Practice.

[7] Ndosi M, Wright-Hughes A, Brown S (2018). Prognosis of the infected diabetic foot ulcer: a 12-month prospective observational study. Diabet Med.

[8] Pemayun TG, Naibaho RM (2017). Clinical profile and outcome of diabetic foot ulcer, a view from tertiary care hospital in Semarang, Indonesia. Diabet Foot Ankle.

[9] Mohammad Zadeh M, Lingsma H, van Neck JW, Vasilic D, van Dishoeck AM (2019). Outcome predictors for wound healing in patients with a diabetic foot ulcer. Int Wound J.

[10] Le TTA, Tran VA, Phan MH (2024). Treatment outcomes, antibiotic selection, and related factors in the management of diabetic foot infections in Vietnam. Endocr Metab Sci.

[11] Vanherwegen AS, Lauwers P, Lavens A, Doggen K, Dirinck E (2023). Sex differences in diabetic foot ulcer severity and outcome in Belgium. PLoS One.

[12] Goh TC, Bajuri MY, C Nadarajah S, Abdul Rashid AH, Baharuddin S, Zamri KS (2020). Clinical and bacteriological profile of diabetic foot infections in a tertiary care. J Foot Ankle Res.

[13] Khan MS, Azam M, Khan MN (2023). Identification of contributing factors, microorganisms and antimicrobial resistance involved in the complication of diabetic foot ulcer treatment. Microb Pathog.

[14] Burande AR, Palekar S, Walke H, Vora TU, Powar SN, Burande MA (2024). Microbiology of diabetic foot (DF) infections: a retrospective analysis to formulate the antibiogram in DF speciality hospital. Int J Med Public Health.

[15] Chen P, Vilorio NC, Dhatariya K (2024). Guidelines on interventions to enhance healing of foot ulcers in people with diabetes (IWGDF 2023 update). Diabetes Metab Res Rev.

[16] Son TK, Toan NH, Thang N (2022). Prediabetes and insulin resistance in a population of patients with heart failure and reduced or preserved ejection fraction but without diabetes, overweight or hypertension. Cardiovasc Diabetol.

[17] Vera-Cruz PN, Palmes PP, Tonogan L, Troncillo AH (2020). Comparison of WIFi, University of Texas and Wagner classification systems as major amputation predictors for admitted diabetic foot patients: a prospective cohort study. Malays Orthop J.

[18] He C, Yang JG, Li YM, Rong J, Du FZ, Yang ZG, Gu M (2014). Comparison of lower extremity atherosclerosis in diabetic and non-diabetic patients using multidetector computed tomography. BMC Cardiovasc Disord.

[19] Lin C, Liu J, Sun H (2020). Risk factors for lower extremity amputation in patients with diabetic foot ulcers: a meta-analysis. PLoS One.

[20] Bekele F, Chelkeba L (2020). Amputation rate of diabetic foot ulcer and associated factors in diabetes mellitus patients admitted to Nekemte referral hospital, western Ethiopia: prospective observational study. J Foot Ankle Res.

[21] Hicks CW, Zhang GQ, Canner JK, Mathioudakis N, Coon D, Sherman RL, Abularrage CJ (2020). Outcomes and predictors of wound healing among patients with complex diabetic foot wounds treated with a dermal regeneration template (Integra). Plast Reconstr Surg.

[22] Meena SP, Badkur M, Lodha M (2024). Impact of multidisciplinary management via special clinic for the outcome of diabetic foot disease: a prospective observational study. J Family Med Prim Care.

[23] Dutra LM, Melo MC, Moura MC, Leme LA, De Carvalho MR, Mascarenhas AN, Novaes MR (2019). Prognosis of the outcome of severe diabetic foot ulcers with multidisciplinary care. J Multidiscip Healthc.

[24] Vahwere BM, Ssebuufu R, Namatovu A (2023). Factors associated with severity and anatomical distribution of diabetic foot ulcer in Uganda: a multicenter cross-sectional study. BMC Public Health.

[25] Bundó M, Vlacho B, Llussà J (2023). Prediction of outcomes in subjects with type 2 diabetes and diabetic foot ulcers in Catalonian primary care centers: a multicenter observational study. J Foot Ankle Res.

[26] Won SH, Chung CY, Park MS (2014). Risk factors associated with amputation-free survival in patient with diabetic foot ulcers. Yonsei Med J.

[27] Chang S, Zhang F, Chen W, Zhou J, Nie K, Deng C, Wei Z (2022). Outcomes of integrated surgical wound treatment mode based on tibial transverse transport for diabetic foot wound. Front Surg.

[28] Lapray M, Petit JM, Fourmont C, Rouland A, Vergès B, Bouillet B (2022). Healing of diabetic foot ulcers is independently associated with the use of angiotensin receptor blockers but not with those of diuretics and angiotensin conversion enzyme inhibitors. Diabetes Metab.

[29] Khan SZ, Montross B, Rivero M, Cherr GS, Harris LM, Dryjski ML, Dosluoglu HH (2020). Angiotensin converting enzyme inhibitors and angiotensin II receptor blockers (ACEI/ARB) are associated with improved limb salvage after Infrapopliteal interventions for critical limb ischemia. Ann Vasc Surg.

[30] Phyo N, Tang W, Kavarthapu V (2021). Medium-term outcomes of multi-disciplinary surgical management of non-ischemic diabetic heel ulcers. J Clin Orthop Trauma.

